# Optimizing Outcomes in Spinal Tumor Surgery: A Meta-analysis Comparing Robotic and Freehand Pedicle Screw Placement

**DOI:** 10.1007/s43465-025-01581-5

**Published:** 2025-10-21

**Authors:** Paweł Łajczak, Anna Łajczak

**Affiliations:** Department of Biophysics, Medical University of Silesia, Katowice, Poland

**Keywords:** Robot-assisted surgery, Pedicle screw placement, Spinal tumors, Metastases, Meta-analysis

## Abstract

**Introduction:**

Accurate pedicle screw placement is critical in spinal tumor and metastasis surgery to ensure stability while minimizing complications. Robot-assisted (RA) techniques have been introduced to improve precision, but their benefits over traditional freehand methods remain uncertain. This study systematically reviews and meta-analyzes the accuracy, safety, and efficiency of RA versus freehand pedicle screw placement in patients with spinal tumors and metastases.

**Methods:**

A systematic search was conducted across five databases. Studies comparing RA and freehand pedicle screw placement in spinal tumor patients were included. Primary outcomes assessed screw accuracy using the Gertzbein–Robbins (GR) classification, while secondary outcomes included operative time, infection rates, and neurological complications. Data were pooled using odds ratios (OR) and mean differences (MD) with a random-effects model.

**Results:**

Three studies were included. RA significantly improved screw placement accuracy (GR A: 76.1% vs. 70.3%, OR 1.34, *p* = 0.023) and reduced minimally misplaced screws (GR B, OR 0.69, *p* = 0.010). The total percentage of clinically acceptable screws (GR A + B) was comparable between groups (90.9% vs. 90.7%, *p* = 0.953). RA surgery significantly reduced operative time (MD − 19.24 min, *p* < 0.01), while infection rates and neurological complications showed no significant differences.

**Conclusion:**

RA pedicle screw placement showed a trend toward a higher rate of perfectly placed screws (GR A), though this was not confirmed by trial sequential analysis. Overall safety profiles were comparable. Further research is needed to evaluate the long-term outcomes and cost-effectiveness of spinal oncology surgery.

## Introduction

Tumors and metastases in the spine area remain a complex challenge for spinal surgeons [1]. The oncological spine patients often require various spinal instrumentation procedures to stabilize the spine, relieve pain, and minimize the risk of nerve root and vessel injury in the surrounding areas [2]. Spine surgeons often opt for pedicle screw placement instrumentation, which is one of the most commonly applied procedures [3]. This technique enables the precise stabilization of areas of interest, providing mechanical support and fusion of the affected vertebrae. While pedicle screw placement remains to be instrumented mostly with traditional freehand-based navigation, several studies suggest an increased risk of misplacement of the pedicle screws in the instrumentation of the spine with this method [4]. Accurate screw placement remains the priority, as improper placement may lead to various complications, including neurological injury, infections, pedicle breach, poor spine stabilization, or the need for reoperation, which leads to additional hospitalization and expenses for the healthcare facility [5]. While countless advantages in technology allowed for more accurate pedicle screw placement, including intraoperative computer-assisted navigation, these techniques are still prone to additional radiation, hand tremors of the surgeon, or the fatigue of the surgical staff during prolonged operations [6]; all of which may affect the screw accuracy. Therefore, more advanced technology is needed to resolve the aforementioned issues.

Robot-assisted (RA) pedicle screw placement has recently been introduced in various surgical tasks, including spine instrumentation [7]. Robotic systems possess mechanized arms and computer-based navigation, and allow for preoperative surgical planning. Various studies in the literature suggest that the use of robotics may provide a more stable screw trajectory, reduced pedicle screw misplacement rate, lower incidence of complications for the patients, shorter surgery time, hospitalization length, or reduced blood loss [8]. Additionally, some reports suggest these systems may be cost-effective [9].

However, while robotic assistance in pedicle screw instrumentation is widely recognized in the literature, the significant majority of meta-analyses or primary studies focus rather on degenerative diseases, scoliosis, or trauma [7–11]. Meanwhile, the data on robotic screw placement in oncological spine surgery remain relatively limited in the literature. Spinal tumors remain a complex challenge for pedicle screw instrumentation due to pathological changes, including bone destruction and deformity, along with the change in biomechanical properties of the spine [12]. The presence of distorted anatomy, bone osteolysis, treatment with radiotherapy, and other oncology-related interventions may significantly impact the pedicle screw placement with freehand guidance. Use of robotic systems could aid spine surgeons in terms of trajectory pre-planning and stabilizing the instrumentation in deformed vertebrae. Therefore, the clinical effectiveness of robotic systems could be heavily influenced by such specific conditions, and proper synthesis is needed to evaluate their effectiveness.

To date, no meta-analysis has undertaken this challenge of analyzing the clinical effectiveness, safety, and clinical outcomes of RA versus conventional freehand pedicle screw instrumentation in oncological patients. The primary objective of this meta-analysis is the evaluation of pedicle screw placement with the use of robotic technology versus the freehand method. Secondary outcomes of this study include operation time, infection rate, and neurological complications. Through this systematic synthesis, this study aims to provide an unbiased, evidence-based assessment of these two methods in the oncological population. The findings of this meta-analysis may further guide clinicians in their decision-making to improve patient safety in the era of rapidly developing computer technology.

## Methods

This systematic review and meta-analysis followed the Preferred Reporting Items for Systematic Reviews and Meta-Analyses (PRISMA) guidelines and the Cochrane Handbook for systematic reviews [13, 14]. The primary aim of this meta-analysis was to compare the accuracy of pedicle screw placement among patients with spinal tumors and metastases with robot-assisted (RA) and freehand surgery.

### Eligibility Criteria

Studies were included if they met the following criteria:

1. Population: Patients diagnosed with primary spinal tumors or spinal metastases undergoing pedicle screw instrumentation procedure.
2. Intervention: Pedicle screw placement with robot-assisted (RA) surgery.
3. Comparator: Pedicle screw placement with the traditional freehand method.
4. Outcomes: At least one of: pedicle screw accuracy, mean operation time, complications (including infection rate, neurological complications, etc.).
5. Study Design: Randomized-controlled trials (RCTs) and observational studies (prospective, retrospective, or case–control studies).

Studies were excluded if they:

1. Did not perform pedicle screw placement procedure (e.g., vertebroplasty, kyphoplasty).
2. Were single-arm studies or did not provide full-text primary data, for example, case reports, case series, conference abstracts, reviews, editorials, letters, or animal studies.
3. Did not provide sufficient data for meta-analysis (e.g., lack of outcomes).

### Search Strategy

Authors performed a systematic search across five electronic databases: PubMed, Embase, Cochrane Library, Web of Science, and Scopus. The systematic search was performed from database inception to January 2025. Keywords and MeSH terms related to robot-assisted, robotic surgery, robotic system, robot, spine, neoplasm, tumor, metastasis, metastases, screw, spinal fixation, spine instrumentation, pedicle fixation, and spine surgery were used (Appendix).

### Study Selection

Screening of the studies was performed by two independent reviewers (blinded to each other's assessments), who screened the titles and abstracts of all imported studies to the screening software. Potentially eligible studies underwent full-text review. Discrepancies during the screening were resolved through mutual discussion with all reviewers. Zotero software was used for the screening process.

### Data Extraction

Two authors extracted data from each study, including:

1. Study characteristics (first author name, publication year, country of origin, design, and duration of the study).
2. Demographic information of the patients (mean age, number of female patients, BMI, tumor diagnosis, and number of patients in each of the groups).
3. Surgical characteristics (robotic device model, total number of screws, and location of the instrumentation).

Outcomes were also extracted by two authors and included:

1. Primary outcome: Accuracy of pedicle screw placement, which was graded according to Gertzbein–Robbins (GR) five-scale classification [Grades (GR) A–E] [15].
2. Secondary outcomes: Mean operation time and complications (infection rates and neurological complications).

Disagreements during data extraction were resolved through mutual discussion with all authors.

### Quality and Risk-of-Bias Assessment

The Risk of Bias in Non-Randomized Studies of Interventions (ROBINS-I) tool was used for quality assessment across seven domains, which are described elsewhere [16]. Risk-of-bias assessment was performed by two authors independently, and each domain was assessed as low, moderate, serious, or critical. Results of the quality assessment were visualized with traffic light plots with the aid of robvis software [17].

### Statistical Analysis

Authors performed a meta-analysis with the use of a meta package in *R* software (v. 4.3.3.) [18]. Categorical endpoints were analyzed with Mantel–Haenszel odds ratio (OR), while continuous with Inverse-Variance mean difference (MD). *P* value < 0.05 was considered statistically significant. The random-effects DerSimonian–Laird model was used for all analyses. Respective effect sizes were reported with 95% confidence intervals (95% CI). Authors assessed the heterogeneity with *I*^2^ statistic, where *I*^2^ > 50% was considered high heterogeneity. The results of the meta-analyses were visualized with forest plots.

### Trial Sequential Analysis (TSA)

Statistically significant outcomes were analyzed with TSA to validate the reliability of the results. The analysis was performed with TSA software [19]. Authors assessed the required information size (RIS) and monitoring boundaries to distinguish between false and true findings. Statistical power was set at 80% and empirical settings were applied for the analysis.

## Results

**Fig. 1:**
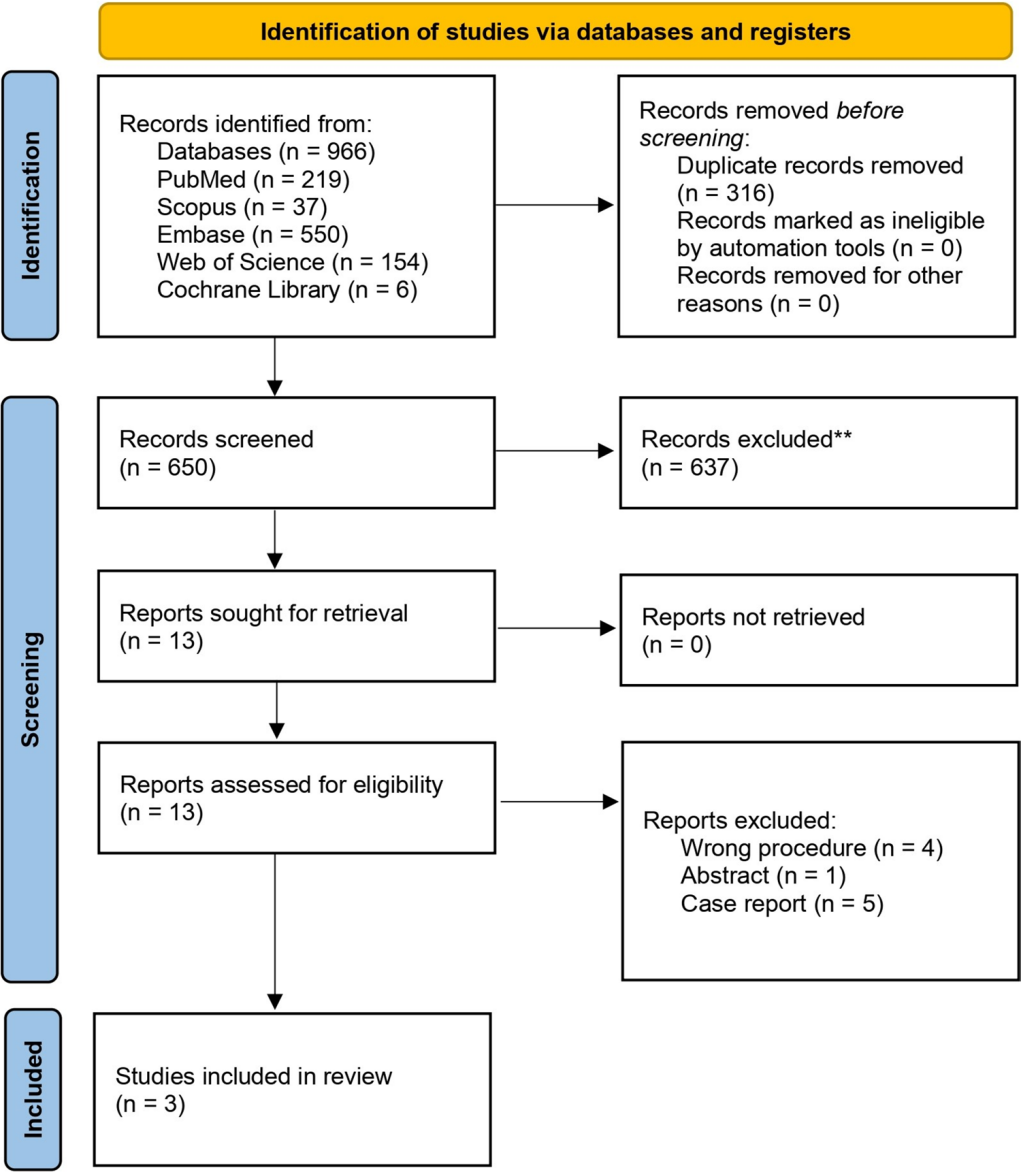
PRISMA flow diagram

A systematic search for the articles of interest was performed from the database inception to January 2025. Five databases were searched, and authors found 219 results from PubMed, 550 from Embase, 6 from Cochrane Library, 154 from Web of Science, and 37 from the Scopus database. Therefore, a total of 966 records were imported for the screening. Zotero screening software identified 316 duplicate entries, which were removed before the proper screening procedure. After this, two authors independently screened the title and abstract of the remaining 650 studies. Two reviewers removed 637 articles that were not relevant to the PICO question of this meta-analysis. All remaining 13 articles were retrieved and underwent independent screening by two authors. Reviewers removed 4 studies, which did not involve pedicle screw placement (including vertebroplasty and kyphoplasty), 5 case reports, and small-sized case series, and 1 conference abstract. Finally, 3 studies were eligible for inclusion after a comprehensive search [20–22]. The detailed search process is visualized in Fig. 1, which depicts the PRISMA flow diagram of the screening.

A total of 3 studies were included in this synthesis. Two studies were from China and one from the Germany–Switzerland collaboration. One study did not specify the robotic system used, one used the TianJi workstation, and the third the Mazor SpineAssist system. A total of 172 patients were included in the meta-analysis, including 83 patients who underwent RA pedicle screw placement and 89 patients who underwent traditional freehand surgery. There were 73 female patients—36 in the RA group and 37 in the freehand cohort. A total of 1324 screws were placed; 618 with RA surgery and 706 with freehand guidance. All studies applied CT scans to perform accuracy assessment of the pedicle screws. The mean age of cohorts ranged from 55.5 to 63.7 years. Tumor diagnosis varied and included lung (43 patients), breast (5 patients), gastrointestinal tract tumors (5 patients), liver (11 patients), lymphoma (9 patients), and multiple myeloma (7 patients). One study did not specify the etiology of the tumor. In general, thoracic and lumbar vertebrae were mostly instrumented in the included studies; however, one study did not specify this in detail. None of the studies experienced failure of the device. Detailed patient and study characteristics are available in Table 1.

**Table 1:** Demographic characteristics of included studies

Study		Wang et al. 2024	Li et al. 2023	Solomiichuk et al. 2017
Institution		Naval Military Medical University, Shanghai, China	Sichuan Academy of Medical Science, Chengdu Sichuan, China	Georg-August-University of Göttingen, Germany; University Hospital Geneva, Université de Genève, Faculté de Médecine, Geneva, Switzerland
Duration		1 st May 2021 to 1 st March 2022	June 2017 and January 2021	June 2009 and June 2015
Robot Model		N/A	TianJi	Mazor SpineAssist
Number of patients	Robot	22	26	35
	Freehand	23	31	35
Number of screws	Robot	169	257	192
	Freehand	176	316	214
Mean age	Robot	61.14 ± 10.00	58.5 ± 8.9	63.7 ± 10.6
	Freehand	55.52 ± 13.52	56.5 ± 9.6	62.2 ± 11.1
Female patients	Robot	11 (50%)	11 (42.31%)	14 (40%)
	Freehand	12 (52.17%)	13 (41.94%)	12 (34.29%)
Number of vertebrae	Robot	N/A	N/A	2.8 ± 1.1
	Freehand	N/A	N/A	3.2 ± 1.2
Mean number of screws	Robot	N/A	N/A	5.5 ± 2.1
	Freehand	N/A	N/A	6.1 ± 2.3
BMI (kg/cm^2^)	Robot	19.56 ± 2.07	20.9 ± 2.9	N/A
	Freehand	22.11 ± 2.76	20.8 ± 2.7	N/A
*Diagnosis (cancer/metastasis)*				
Lung	Robot	13	8	N/A
	Freehand	12	10	N/A
Breast	Robot	N/A	2	N/A
	Freehand	N/A	3	N/A
GI tract	Robot	0	2	N/A
	Freehand	1	2	N/A
Liver	Robot	2	2	N/A
	Freehand	3	4	N/A
Lymphoma	Robot	N/A	4	N/A
	Freehand	N/A	5	N/A
Multiple myeloma	Robot	3	N/A	N/A
	Freehand	4	N/A	N/A
Prostate	Robot	2	1	N/A
	Freehand	1	1	N/A
Renal carcinoma	Robot	1	2	N/A
	Freehand	1	2	N/A
Parotid carcinoma	Robot	1	N/A	N/A
	Freehand	1	N/A	N/A
Other	Robot	N/A	5	N/A
	Freehand	N/A	4	N/A
*Operated vertebrae*				
Thoracic	Robot	14	11	N/A
	Freehand	14	13	N/A
Thoracolumbar	Robot	N/A	9	N/A
	Freehand	N/A	12	N/A
Lumbar	Robot	8	6	N/A
	Freehand	9	6	N/A

The accuracy of pedicle screw accuracy varied. The detailed raw pedicle screw accuracy results are represented in Table 2.

**Table 2:** Results of the pedicle screw accuracy

Study	Screws (total)		GR A		GR B		GR C		GR D		GR E	
Wang et al. 2024	169	176	149	140	20	36	0	0	0	0	0	0
Li et al. 2023	257	316	192	220	39	65	23	25	3	5	0	1
Solomiichuk et al. 2017	192	214	129	136	33	43	10	18	14	9	6	8

### Screw Placement Accuracy

**Fig. 2:**
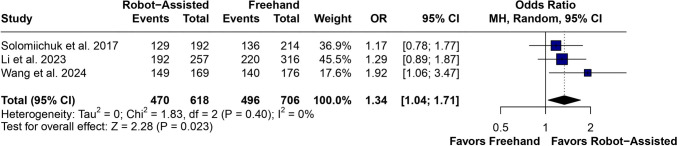
Forest plot for GR A (no malposition) screws

The screw placement without malposition (GR A) was reported in 3 studies. A total of 470/618 (76.1%) screws in the RA group and 496/706 (70.3%) screws in the freehand group were placed without malposition (GR A). This difference was statistically significant (OR 1.34 (95% CI: 1.04 to 1.71), *p* = 0.023; Fig. 2). There was no inconsistency in the analysis (*I*^2^ = 0%, *p* = 0.40).

There were 92/618 (14.9%) GR B screws in the RA cohort and 144/706 (20.4%) GR B screws in the traditional group. The number of screws with minimal malposition (GR B) was significantly lower in the RA group (OR 0.69 (95% CI 0.51–0.91), *p* = 0.010). There was zero inconsistency (*I*^2^ = 0%, *p* = 0.51).

**Fig. 3:**
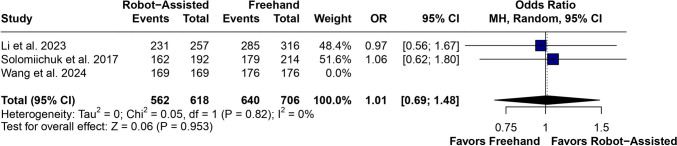
Forest plot for clinically acceptable screws (sum of GR A and GR B)

The number of clinically acceptable screws (sum of GR A and GR B) was 562/618 (90.9%) in the RA group and 640/706 (90.7%) in the freehand group. Authors found no significant difference between the two groups [OR 1.01 (95% CI 0.69 to 1.48), *p* = 0.953; Fig. 3]. No inconsistency was observed (*I*^2^ = 0%, *p* = 0.82).

There were 33/618 (5.3%) GR C screws in the RA group and 43/706 (6.1%) in the freehand group. The incidence of GR C screws was not significantly different between RA and freehand groups [OR 0.88 (95% CI 0.47 to 1.64), *p* = 0.680]. Some inconsistency was observed (*I*^2^ = 39%, *p* = 0.20).

**Fig. 4:**
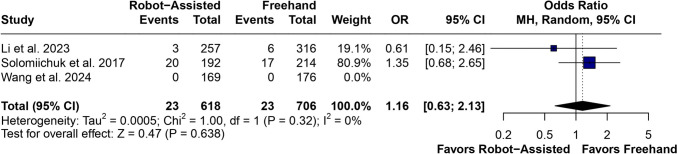
Forest plot for sum of GR D and GR E screws

The rate of severely misplaced screws (> 4 mm, sum of GR D and GR E) was low in both groups—23/618 (3.7%) in RA and 23/706 (3.3%) in freehand. The incidence of severely misplaced screws was not significant (OR 1.16 (95% CI 0.63 to 2.13), *p* = 0.638; Fig. 4). No inconsistency was observed in the analysis (*I*^2^ = 0%, *p* = 0.32).

### Infections

**Fig. 5:**
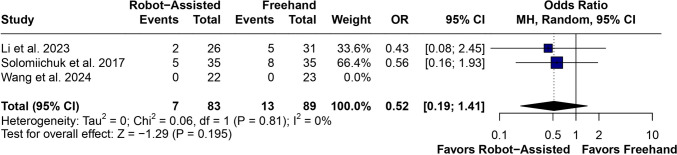
Forest plot for infection rate

The infection rate was reported in all 3 studies. There was no statistically significant difference between two groups [OR 0.52 (95% CI: 0.19 to 1.41), *p* = 0.195; Fig. 5]. No inconsistency was observed in the analysis (*I*^2^ = 0%, *p* = 0.81).

### Neurological Injury

**Fig. 6:**
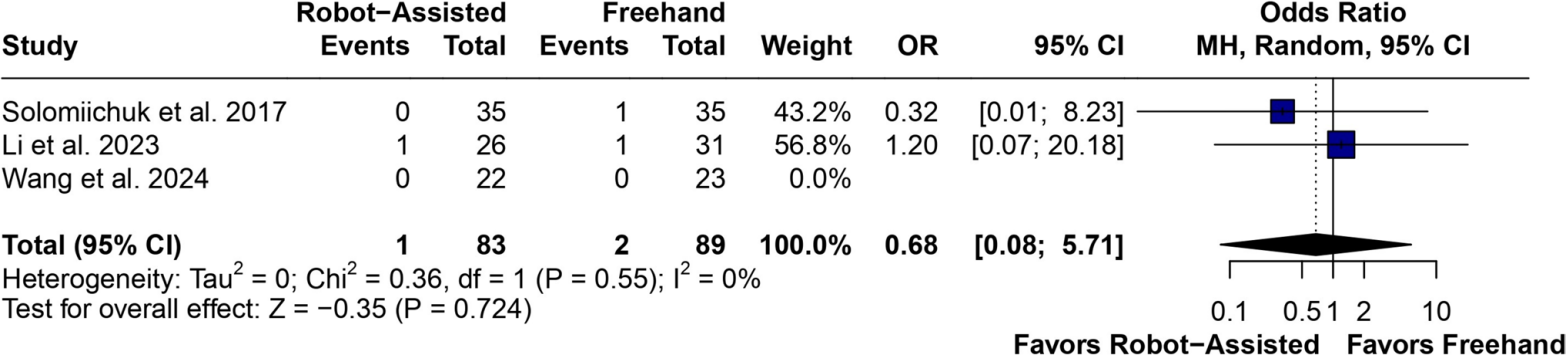
Forest plot for neurological injury

There was no significant difference in terms of neurological injury between two groups (OR 0.68 (95% CI 0.08 to 5.71), *p* = 0.724; Fig. 6). No inconsistency was observed in the analysis (*I*^2^ = 0%, *p* = 0.55). No other significant complications occurred among the patients in both groups.

### Operation Time

**Fig. 7:**
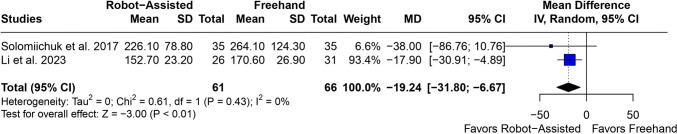
Forest plot for operation time

The operation time was significantly lower with the use of robotic surgery (MD − 19.24 (95% CI − 31.80 to − 6.67), *p* < 0.01; Fig. 7). No inconsistency was observed in the analysis (*I*^2^ = 0%, *p* = 0.43).

### Trial Sequential Analysis

**Fig. 8:**
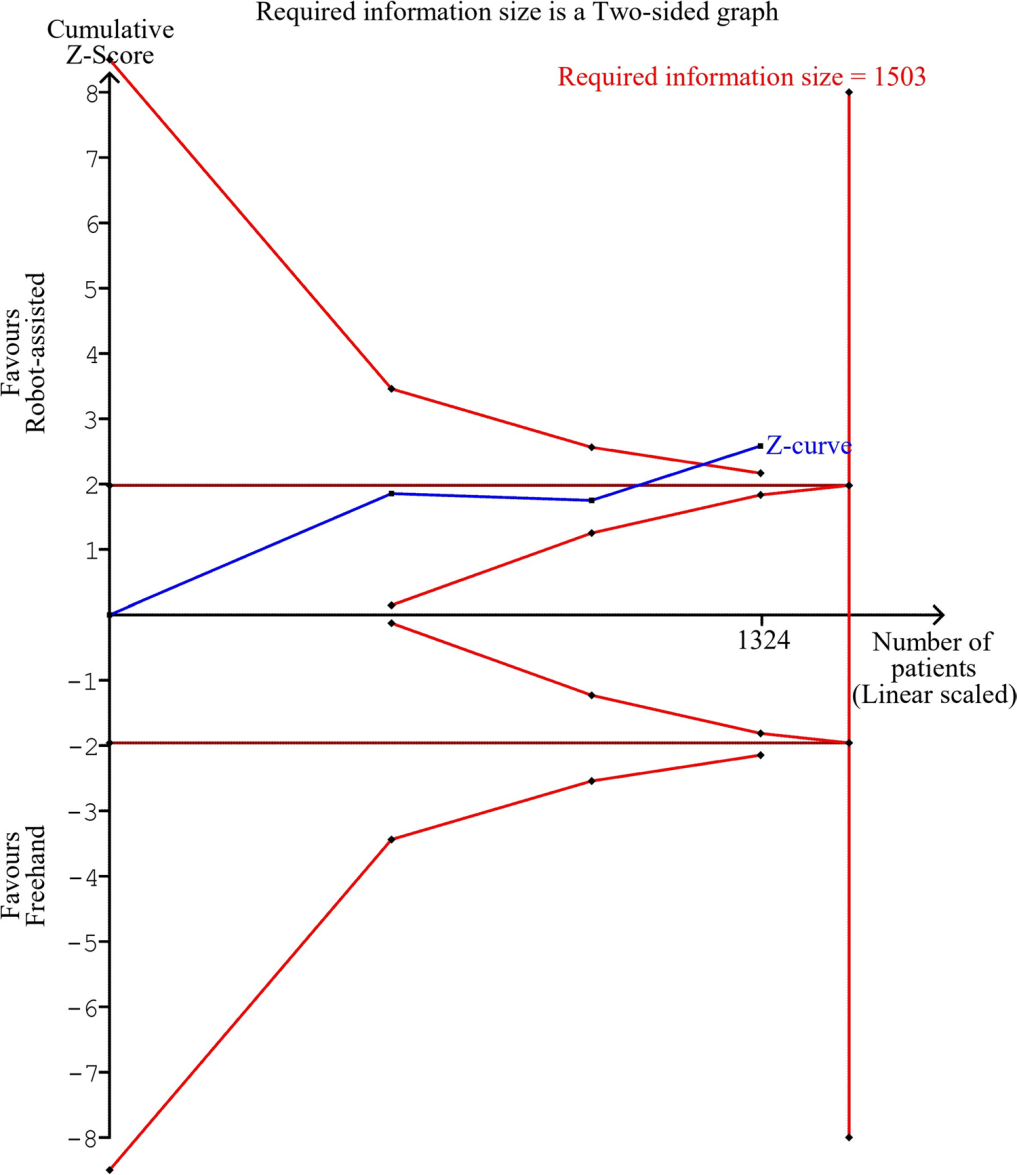
TSA for GR B screws

**Fig. 9:**
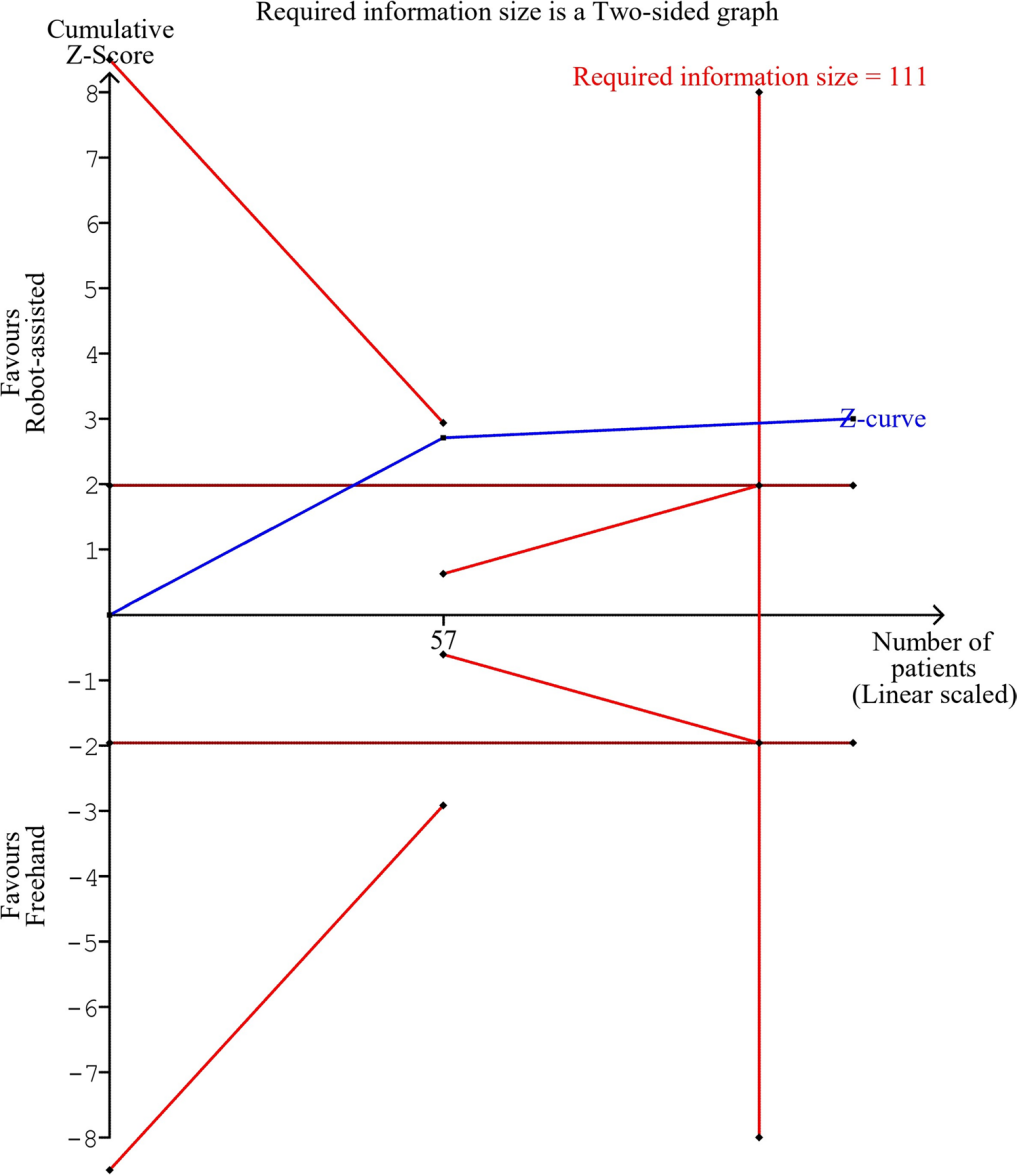
TSA for operation time

Trial sequential analysis was performed to validate the significant outcomes of this meta-analysis. In the incidence of GR B screws (Fig. 8) and operation time (Fig. 9), the Z-curve crossed the significance boundary with the required information size or monitoring boundary, yielding a true-positive result. In GR A screws, Z-curve did not cross the required information size and monitoring (superiority) boundary, yielding a false-positive result.

### Quality Assessment

**Fig. 10:**
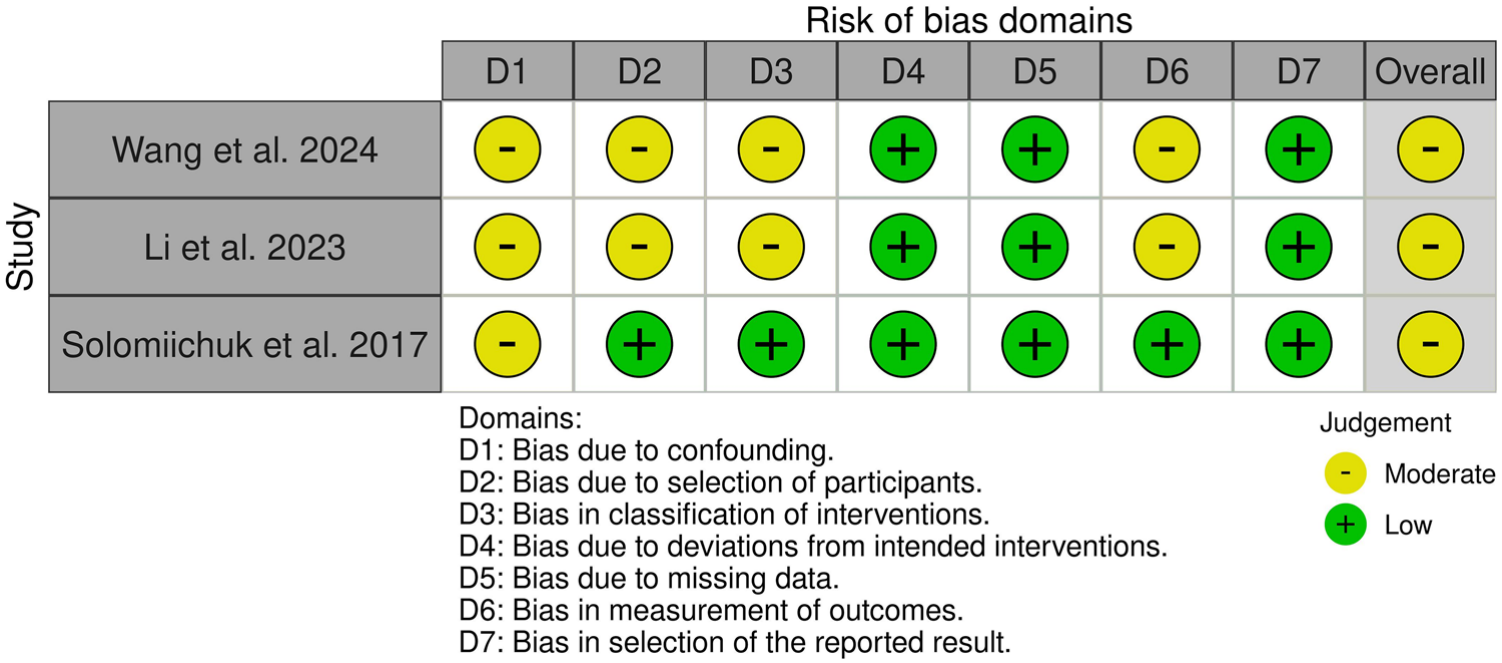
Quality assessment results

Risk-of-bias assessment was performed by two authors independently, who assessed seven domains in the ROBINS-I tool. All studies were graded with moderate bias concerns. Most of the risk was found in confounding, selection of participants, classification of interventions, and measurement of the outcomes. Detailed results of the quality assessment are available in Fig. 10.

## Discussion

This systematic review and pairwise meta-analysis aimed to compare the effectiveness of RA and conventional freehand surgery in pedicle screw instrumentation among oncological patients with spinal tumors and metastases. The pooled meta-analysis showed significantly higher odds of GR A screws in the RA group compared to traditional freehand technique (76.1% in RA vs. 70.3% in FH, OR 1.34, *p* = 0.023). Although our primary meta-analysis indicated a significantly higher odds of an ideal (GR A) screw placement, this finding must be interpreted with caution. The TSA revealed this result did not reach the required information size to be considered a definitive true positive. This suggests that while RA technology shows promise for improving precision, the current body of evidence is insufficient to conclusively support this claim. Moreover, the total number of clinically acceptable screws (GR A + GR B) remained comparable between the two groups (90.9% vs. 90.7%), and meta-analysis did not indicate a significant difference.

Besides pedicle screw accuracy, the use of robotic surgery led to a significant reduction in mean surgery time (MD − 19.24 min, *p* < 0.01). However, authors found no significant differences in complication rates; more specifically, there was no reduction in infection rates or neurological complications using robotics. Findings of the meta-analysis show that while RA surgery may show a trend for improved accuracy, the safety remains comparable to the traditional freehand methods.

Accurate pedicle screw placement remains a paramount task in oncological spine instrumentation, as various factors, including tumor presence, radiotherapy surgery, or previous oncological or spinal procedures, may influence the biomechanical and tissue properties of the spine, making the task relatively challenging. The results of this meta-analysis indicate that the use of robotic surgery showed a trend toward improved accuracy. This could be a result of computer-assisted navigation, preoperative planning, and properties of robotic arms, which reduce hand tremors, optimize trajectory, and are prone to fatigue. The robotic platform provides a stable tool for precise screw placement with real-time feedback, warning systems, and monitoring of tools and screws. This advantage might be especially beneficial among the novice residents of spine surgery, who might achieve faster mastery of screw placement. For example, in the study conducted by Wang and colleagues, residents who were trained with the use of a robotic platform achieved proficiency faster compared to residents who were trained with traditional methods. In this study, residents trained with RA surgery needed 121 pedicle screws to achieve proficiency, while residents in the traditional group needed 138.

However, while the robotic surgery provided higher odds for fully accurate screws, the overall percentage of clinically acceptable screws (sum of GR A and GR B screws) remained comparable between the two groups. These findings suggest that while RA indeed improves the accuracy of the screws, the overall effectiveness of both techniques remains highly effective. This was confirmed, as the incidence of severely misplaced screws (sum of GR D and GR E screws) remained relatively low in both cohorts (3.7% vs. 3.3%), submitting that both methods remain highly effective in spine instrumentation procedures.

Moreover, the results of this meta-analysis indicate that the use of robotic surgery leads to a significant reduction in mean operation time compared to traditional methods. The surgical time was lower with RA assistance, which might be a result of an improved workflow, advanced preoperative navigation, and computer-aided trajectory planning, along with reduced intraoperative adjustments. The reduced operation time might be especially valuable among patients with spinal tumors and metastases, as the prolonged operation time may increase the total bleeding volume, possible infections, or complications related to anesthesia. For example, in the Li et al.’s study, the RA surgery reduced intraoperative blood loss by 214.347 ml (*p* = 0.023) compared to the conventional freehand surgery [22]. Furthermore, while not analyzed in this study, this could also reduce the need for additional fluoroscopy shots or manual corrections, further streamlining the surgery process.

Interestingly, the complications, including infections and neurological injury events, were comparable between the RA and freehand groups, and authors did not find any significant differences between these two. While RA surgery improved accurate pedicle screw placement, this advantage did not directly translate into a reduction in complication rate. These findings suggest that various factors, including preoperative conditions of the patient, sterile environment, and perioperative control, might be more relevant in terms of control of infections, than the use of the RA system itself. Similarly, neurological safety was comparable between the two cohorts, mainly due to the low rate of severe pedicle screw misplacement.

The results of this meta-analysis suggest several points for clinical practice. The trend in improved screw accuracy might be especially beneficial in patients with severe stages of tumors and metastases, where vertebral destruction, neoplasm-related deformities, or other invasive interventions were performed. The implementation of robotic technology might play a crucial role in patients with less reliable anatomical landmarks or different biomechanical properties of the spine due to tumor changes, especially benefiting novice spine surgeons. Furthermore, postoperative morbidity might be influenced due to reduced total operation time, which was significantly shorter with RA surgery compared to the freehand group.

However, several aspects should be considered before the full adoption of RA workstations. First, authors observed that the number of clinically acceptable screws was comparable between the two groups. Therefore, the acquisition of robotic systems should be guided by various factors, especially taking into consideration available financial resources and various patient-specific factors. Robotic technology should ideally remain a cost-effective tool to optimize the workflow of the healthcare institution.

The overall effectiveness of RA surgery remains under the high influence of the learning curve and accessibility of the system. Regarding the economic factors, Li et al. found no significant difference between RA and freehand surgery in terms of hospital expenses (*p* = 0.392) [22]. Notably, they observed shorter hospital stays (MD − 4.89, *p* < 0.001) and lower ODI scores (MD − 9.59, *p* < 0.01) compared to freehand surgery patients. The widespread adoption of surgical robots remains limited mainly due to high initial purchase costs. These might further increase due to maintenance, training, and equipment expenses. The cost-effectiveness could be especially visible in high-volume surgical centers, where RA workstations could optimize the overall workflow.

There are several limitations of this meta-analysis, which should be considered during the interpretation of the findings. First, this review included only 3 studies, which limits the overall sample size and power of the meta-analysis. Only 172 patients were included in the review; therefore, the conclusions of this meta-analysis should be considered as preliminary (exploratory), rather than definitive, as more randomized studies are needed to validate the findings. Additionally, the sample size and power of the accuracy of the screws meta-analysis were inflated, as the number of screws exceeded the number of patients (1324 vs. 172).

Moreover, differences in robotic platforms, oncological diagnoses, and surgical approaches were observed, influencing the inconsistency in the outcomes. Additionally, studies varied in terms of operator experience and imaging protocols during the surgery. These factors could impact pedicle screw accuracy, variability in terms of screw grading assessment, or length of the procedure, as more experienced surgeons could perform this instrumentation faster than less experienced ones in oncological spines. Authors observed a moderate risk of bias concerns in all included studies, which could influence the quality of the meta-analysis. Finally, all outcomes focused on short-term follow-up outcomes, rather than long-term, as studies mostly provided screw placement accuracy and perioperative outcomes.

Therefore, larger randomized trials with greater sample sizes, focusing on long-term patient outcomes, should be performed. Additionally, more cost-effectiveness analyses could establish whether the use of RA surgery for oncological patients is economically beneficial over freehand surgery.

## Conclusions

To conclude, RA pedicle screw placement showed a trend toward a higher rate of perfectly placed screws (GR A), though this was not confirmed by trial sequential analysis. Overall safety profiles were comparable. Policymakers should take into consideration various economic factors before the implementation of such devices. Furthermore, more long-term research is needed to fully validate the clinical effectiveness of robotic surgery in spinal oncology patients.

## Data Availability

Data used in this study is available in the respective references.

## Appendix: Search Strategy

Cochrane: (robot-assisted OR robotic surgery OR robotic-assisted OR robotic technology OR robotic system OR surgical robot OR robot) AND (spine AND (neoplasm OR neoplasms OR tumor OR metastasis OR metastases)) AND (pedicle OR screw OR spinal fixation OR spine instrumentation OR pedicle fixation OR spine surgery) in Title Abstract Keyword.

Scopus: TITLE-ABS-KEY ((robot-assisted OR robotic AND surgery OR robotic-assisted OR robotic AND technology OR robotic AND system OR surgical AND robot OR robot) AND (spine AND (neoplasm OR neoplasms OR tumor OR metastasis OR metastases)) AND (pedicle OR screw OR spinal AND fixation OR spine AND instrumentation OR pedicle AND fixation OR spine AND surgery)).

Embase: ('robot assisted' OR 'robotic surgery'/exp OR 'robotic surgery' OR (robotic AND ('surgery'/exp OR surgery)) OR 'robotic assisted' OR 'robotic technology' OR (robotic AND ('technology'/exp OR technology)) OR 'robotic system' OR (robotic AND system) OR 'surgical robot'/exp OR 'surgical robot' OR (surgical AND ('robot'/exp OR robot)) OR 'robot'/exp OR robot) AND ('spine'/exp OR spine) AND ('neoplasm'/exp OR neoplasm OR 'neoplasms'/exp OR neoplasms OR 'tumor'/exp OR tumor OR 'metastasis'/exp OR metastasis OR 'metastases'/exp OR metastases) AND ('pedicle'/exp OR pedicle OR 'screw'/exp OR screw OR 'spinal fixation' OR (spinal AND ('fixation'/exp OR fixation)) OR 'spine instrumentation' OR (('spine'/exp OR spine) AND ('instrumentation'/exp OR instrumentation)) OR 'pedicle fixation' OR (('pedicle'/exp OR pedicle) AND ('fixation'/exp OR fixation)) OR 'spine surgery'/exp OR 'spine surgery' OR (('spine'/exp OR spine) AND ('surgery'/exp OR surgery))).

Web of Science: (robot-assisted OR robotic surgery OR robotic-assisted OR robotic technology OR robotic system OR surgical robot OR robot) AND (spine AND (neoplasm OR neoplasms OR tumor OR metastasis OR metastases)) AND (pedicle OR screw OR spinal fixation OR spine instrumentation OR pedicle fixation OR spine surgery).

Pubmed: (robot-assisted[All Fields] OR (robotic surgical procedures[MeSH Terms] OR (robotic[All Fields] AND surgical[All Fields] AND procedures[All Fields]) OR robotic surgical procedures[All Fields] OR (robotic[All Fields] AND surgery[All Fields]) OR robotic surgery[All Fields]) OR robotic-assisted[All Fields] OR ((robot[All Fields] OR robot s[All Fields] OR robotically[All Fields] OR robotics[MeSH Terms] OR robotics[All Fields] OR robotic[All Fields] OR robotization[All Fields] OR robotized[All Fields] OR robots[All Fields]) AND (technology[MeSH Terms] OR technology[All Fields] OR technologies[All Fields] OR technology s[All Fields])) OR ((robot[All Fields] OR robot s[All Fields] OR robotically[All Fields] OR robotics[MeSH Terms] OR robotics[All Fields] OR robotic[All Fields] OR robotization[All Fields] OR robotized[All Fields] OR robots[All Fields]) AND (system[All Fields] OR system s[All Fields] OR systems[All Fields])) OR ((surgical procedures, operative[MeSH Terms] OR (surgical[All Fields] AND procedures[All Fields] AND operative[All Fields]) OR operative surgical procedures[All Fields] OR surgical[All Fields] OR surgically[All Fields] OR surgicals[All Fields]) AND (robot[All Fields] OR robot s[All Fields] OR robotically[All Fields] OR robotics[MeSH Terms] OR robotics[All Fields] OR robotic[All Fields] OR robotization[All Fields] OR robotized[All Fields] OR robots[All Fields])) OR (robot[All Fields] OR robot s[All Fields] OR robotically[All Fields] OR robotics[MeSH Terms] OR robotics[All Fields] OR robotic[All Fields] OR robotization[All Fields] OR robotized[All Fields] OR robots[All Fields])) AND ((spine[MeSH Terms] OR spine[All Fields] OR spines[All Fields] OR spine s[All Fields]) AND (neoplasm s[All Fields] OR neoplasms[MeSH Terms] OR neoplasms[All Fields] OR neoplasm[All Fields] OR (neoplasm s[All Fields] OR neoplasms[MeSH Terms] OR neoplasms[All Fields] OR neoplasm[All Fields]) OR (cysts[MeSH Terms] OR cysts[All Fields] OR cyst[All Fields] OR neurofibroma[MeSH Terms] OR neurofibroma[All Fields] OR neurofibromas[All Fields] OR tumor s[All Fields] OR tumoral[All Fields] OR tumorous[All Fields] OR tumour[All Fields] OR neoplasms[MeSH Terms] OR neoplasms[All Fields] OR tumor[All Fields] OR tumour s[All Fields] OR tumoural[All Fields] OR tumourous[All Fields] OR tumours[All Fields] OR tumors[All Fields]) OR (metastasi[All Fields] OR neoplasm metastasis[MeSH Terms] OR (neoplasm[All Fields] AND metastasis[All Fields]) OR neoplasm metastasis[All Fields] OR metastasis[All Fields]) OR (metastasation[All Fields] OR metastasic[All Fields] OR metastasing[All Fields] OR metastasise[All Fields] OR metastasised[All Fields] OR metastasises[All Fields] OR metastasising[All Fields] OR metastasization[All Fields] OR metastasizes[All Fields] OR metastasizing[All Fields] OR neoplasm metastasis[MeSH Terms] OR (neoplasm[All Fields] AND metastasis[All Fields]) OR neoplasm metastasis[All Fields] OR metastase[All Fields] OR metastases[All Fields] OR metastasize[All Fields] OR metastasized[All Fields]))) AND (pedicle[All Fields] OR pedicle s[All Fields] OR pedicled[All Fields] OR pedicles[All Fields] OR (bone screws[MeSH Terms] OR (bone[All Fields] AND screws[All Fields]) OR bone screws[All Fields] OR screw[All Fields] OR screw s[All Fields] OR screwed[All Fields] OR screwing[All Fields] OR screws[All Fields]) OR ((spinal[All Fields] OR spinalization[All Fields] OR spinalized[All Fields] OR spinally[All Fields] OR spinals[All Fields]) AND (fixate[All Fields] OR fixated[All Fields] OR fixates[All Fields] OR fixating[All Fields] OR fixation[All Fields] OR fixational[All Fields] OR fixations[All Fields] OR fixator[All Fields] OR fixator s[All Fields] OR fixators[All Fields])) OR ((spine[MeSH Terms] OR spine[All Fields] OR spines[All Fields] OR spine s[All Fields]) AND (instrumentation[MeSH Subheading] OR instrumentation[All Fields] OR instrumentation s[All Fields] OR instrumentational[All Fields] OR instrumentations[All Fields] OR instrumention[All Fields])) OR ((pedicle[All Fields] OR pedicle s[All Fields] OR pedicled[All Fields] OR pedicles[All Fields]) AND (fixate[All Fields] OR fixated[All Fields] OR fixates[All Fields] OR fixating[All Fields] OR fixation[All Fields] OR fixational[All Fields] OR fixations[All Fields] OR fixator[All Fields] OR fixator s[All Fields] OR fixators[All Fields])) OR ((spine[MeSH Terms] OR spine[All Fields] OR spines[All Fields] OR spine s[All Fields]) AND (surgery[MeSH Subheading] OR surgery[All Fields] OR surgical procedures, operative[MeSH Terms] OR (surgical[All Fields] AND procedures[All Fields] AND operative[All Fields]) OR operative surgical procedures[All Fields] OR general surgery[MeSH Terms] OR (general[All Fields] AND surgery[All Fields]) OR general surgery[All Fields] OR surgery s[All Fields] OR surgerys[All Fields] OR surgeries[All Fields]))).

## References

[1] Goodwin, M. L., Buchowski, J. M., Schwab, J. H., & Sciubba, D. M. (2022). Spinal tumors: Diagnosis and treatment. *Journal of The American Academy of Orthopaedic Surgeons,**30*(17), e1106. 10.5435/JAAOS-D-21-00710 35984082 10.5435/JAAOS-D-21-00710

[2] Spivak, J. M., & Balderston, R. A. (1994). Spinal instrumentation. *Current Opinion in Rheumatology,**6*(2), 187–194. 10.1097/00002281-199403000-00012 8024965 10.1097/00002281-199403000-00012

[3] Perna, F., Borghi, R., Pilla, F., Stefanini, N., Mazzotti, A., & Chehrassan, M. (2016). Pedicle screw insertion techniques: An update and review of the literature. *Musculoskeletal Surgery,**100*(3), 165–169. 10.1007/s12306-016-0438-8 27866324 10.1007/s12306-016-0438-8

[4] Alomari, S., Lubelski, D., Lehner, K., et al. (2023). Safety and accuracy of freehand pedicle screw placement and the role of intraoperative O-arm: A single institution experience. *Spine*. 10.1097/brs.0000000000004497 36190990 10.1097/BRS.0000000000004497

[5] Yamada, T., Hasegawa, T., Yamato, Y., et al. (2022). Characteristics of pedicle screw misplacement using freehand technique in degenerative scoliosis surgery. *Archives of Orthopaedic and Trauma Surgery,**143*(4), 1861–1867. 10.1007/s00402-022-04380-x 35194658 10.1007/s00402-022-04380-x

[6] Sembrano, J. N., Yson, S. C., & Theismann, J. (2019). Computer navigation in minimally invasive spine surgery. *Current Reviews In Musculoskeletal Medicine,**12*(4), 415–424. 10.1007/s12178-019-09577-z 31701412 10.1007/s12178-019-09577-z PMC6942055

[7] Łajczak, P., Łajczak, A., Buczkowski, S., et al. (2025). An early evaluation of robot-assisted and conventional techniques for posterior approach atlantoaxial displacement instrumentation - a systematic review and meta-analysis. *Neurosurgical Review,**48*(1), 105. 10.1007/s10143-025-03256-z 39883207 10.1007/s10143-025-03256-z

[8] Lopez, I. B., Benzakour, A., Mavrogenis, A., Benzakour, T., Ahmad, A., & Lemée, J. M. (2022). Robotics in spine surgery: Systematic review of literature. *International Orthopaedics,**47*(2), 447–456. 10.1007/s00264-022-05508-9 35849162 10.1007/s00264-022-05508-9

[9] Menger, R. P., Savardekar, A. R., Farokhi, F., & Sin, A. (2018). A cost-effectiveness analysis of the integration of robotic spine technology in spine surgery. *Neurospine,**15*(3), 216–224. 10.14245/ns.1836082.041 30157583 10.14245/ns.1836082.041 PMC6226125

[10] Luengo-Matos, S., Sánchez-Gómez, L. M., Hijas-Gómez, A. I., García-Carpintero, E. E., Ballesteros-Massó, R., & Polo-deSantos, M. (2022). Efficacy and safety of robotic spine surgery: Systematic review and meta-analysis. *Journal of Orthopaedics and Traumatology: Official Journal of the Italian Society of Orthopaedics and Traumatology,**23*(1), 49. 10.1186/s10195-022-00669-0 36242652 10.1186/s10195-022-00669-0 PMC9569281

[11] Chumnanvej, S., Pillai, B. M., Suthakorn, J., & Chumnanvej, S. (2024). Revised in-depth meta-analysis on the efficacy of robot-assisted versus traditional free-hand pedicle screw insertion. *Laparoscopic, Endoscopic and Robotic Surgery*. 10.1016/j.lers.2024.08.002

[12] Salvatore, G., Berton, A., Giambini, H., et al. (2018). Biomechanical effects of metastasis in the osteoporotic lumbar spine: A finite element analysis. *BMC Musculoskeletal Disorders*. 10.1186/s12891-018-1953-6 29402261 10.1186/s12891-018-1953-6 PMC5799979

[13] Higgins, J. P. T., Thomas, J., Chandler, J., et al. (Eds.). (2019). *Cochrane Handbook for Systematic Reviews of Interventions*. Wiley. 10.1002/14651858.ED000142 PMC10284251 31643080

[14] Page, M. J., McKenzie, J. E., Bossuyt, P. M., et al. (2021). The PRISMA 2020 statement: An updated guideline for reporting systematic reviews. *British Medical Journal*. 10.1136/bmj.n71 33782057 10.1136/bmj.n71 PMC8005924

[15] Gertzbein, S. D., & Robbins, S. E. (1990). Accuracy of pedicular screw placement in vivo. *Spine,**15*(1), 11–14. 10.1097/00007632-199001000-00004 2326693 10.1097/00007632-199001000-00004

[16] Sterne, J. A., Hernán, M. A., Reeves, B. C., et al. (2016). ROBINS-I: A tool for assessing risk of bias in non-randomised studies of interventions. *BMJ,**355*(355), Article i4919. 10.1136/bmj.i4919 27733354 10.1136/bmj.i4919 PMC5062054

[17] McGuinness, L. A., & Higgins, J. P. T. (2020). Risk-of-bias VISualization (robvis): An R package and Shiny web app for visualizing risk-of-bias assessments. *Research Synthesis Methods*. 10.1002/jrsm.1411 32336025 10.1002/jrsm.1411

[18] Balduzzi, S., Rücker, G., & Schwarzer, G. (2019). How to perform a meta-analysis with R: A practical tutorial. *Evidence-Based Mental Health,**22*(4), 153–160. 10.1136/ebmental-2019-300117 31563865 10.1136/ebmental-2019-300117 PMC10231495

[19] Trial Sequential Analysis (TSA) [Computer program]. Version 0.9.5.10 Beta. The Copenhagen Trial Unit, Centre for Clinical Intervention Research, The Capital Region, Copenhagen University Hospital – Rigshospitalet, 2021. https://ctu.dk/tsa/downloads/

[20] Wang, P., Xin, Y., Zhou, S., et al. (2024). Efficacy of computer-assisted robotic based clinical training program for spinal oncology education on pedicle screw placement. *Journal of Robotic Surgery*. 10.1007/s11701-023-01804-7 38564025 10.1007/s11701-023-01804-7 PMC10987351

[21] Solomiichuk, V., Fleischhammer, J., Molliqaj, G., et al. (2017). Robotic versus fluoroscopy-guided pedicle screw insertion for metastatic spinal disease: A matched-cohort comparison. *Neurosurgical Focus,**42*(5), E13. 10.3171/2017.3.focus1710 28463620 10.3171/2017.3.FOCUS1710

[22] Li, J., Lin, S., Tang, L., et al. (2023). Effectiveness of robot-guided percutaneous fixation and decompression via small incision for advanced thoracolumbar metastases. *Chinese Journal of Reparative and Reconstructive Surgery*. 10.7507/1002-1892.202305040 37718424 10.7507/1002-1892.202305040 PMC10505639

